# *In-vivo* Imaging of Mitochondrial Depolarization of Myocardium With Positron Emission Tomography and a Proton Gradient Uncoupler

**DOI:** 10.3389/fphys.2020.00491

**Published:** 2020-05-15

**Authors:** Nathaniel M. Alpert, Matthieu Pelletier-Galarneau, Sally Ji Who Kim, Yoann Petibon, Tao Sun, Karla M. Ramos-Torres, Marc D. Normandin, Georges El Fakhri

**Affiliations:** ^1^Gordon Center for Medical Imaging, Department of Radiology, Massachusetts General Hospital, Harvard Medical School, Boston, MA, United States; ^2^Department of Medical Imaging, Montreal Heart Institute, Montreal, QC, Canada

**Keywords:** mitochondrial membrane potential, tissue membrane potential, tetraphenylphosphonium, positron emission tomography, BAM15

## Abstract

**Background:**

We recently reported a method using positron emission tomography (PET) and the tracer ^18^F-labeled tetraphenylphosphonium (^18^F-TPP^+^) for mapping the tissue (i.e., cellular plus mitochondrial) membrane potential (ΔΨ_T_) in the myocardium. The purpose of this work is to provide additional experimental evidence that our methods can be used to observe transient changes in the volume of distribution for ^18^F-TPP^+^ and mitochondrial membrane potential (ΔΨ_m_).

**Methods:**

We tested these hypotheses by measuring decreases of ^18^F-TPP^+^ concentration elicited when a proton gradient uncoupler, BAM15, is administered by intracoronary infusion during PET scanning. BAM15 is the first proton gradient uncoupler shown to affect the mitochondrial membrane without affecting the cellular membrane potential. Preliminary dose response experiments were conducted in two pigs to determine the concentration of BAM15 infusate necessary to perturb the ^18^F-TPP^+^ concentration. More definitive experiments were performed in two additional pigs, in which we administered an intravenous bolus plus infusion of ^18^F-TPP^+^ to reach secular equilibrium followed by an intracoronary infusion of BAM15.

**Results:**

Intracoronary BAM15 infusion led to a clear decrease in ^18^F-TPP^+^ concentration, falling to a lower level, and then recovering. A second BAM15 infusion reduced the ^18^F-TPP^+^ level in a similar fashion. We observed a maximum depolarization of 10 mV as a result of the BAM15 infusion.

**Summary:**

This work provides evidence that the total membrane potential measured with ^18^F-TPP^+^ PET is sensitive to temporal changes in mitochondrial membrane potential.

## Introduction

The mitochondrial membrane potential ΔΨ_m_ represents the energy stored in the electric field of the mitochondrion that is used to convert ADP to ATP. Importantly, mitochondrial membrane potential is normally regulated within rather narrow bounds (Kadenbach et al., 2011). Whenever the mitochondrial membrane potential is abnormally high or low, additional reactive oxygen species (ROS) are generated, leading to an environment toxic to mitochondria (Cui et al., 2012; Suski et al., 2018). Mitochondrial dysfunction has been shown to play a critical role in several pathologies, including heart failure (Kadenbach et al., 2011), ventricular arrhythmia (Aon et al., 2003; O’rourke et al., 2005; Rutledge and Dudley, 2013), myopathy (Widlansky et al., 2010; Walters et al., 2012; Pfeffer and Chinnery, 2013), diabetes (Green et al., 2004; Widlansky et al., 2010; De Felice and Ferreira, 2014), and cancer proliferation (Momcilovic et al., 2019). As mitochondrial membrane potential is an essential determinant of mitochondrial functional status (Chen, 1988; Duchen, 2004; O’rourke, 2016), its noninvasive quantitative mapping might provide a new biomarker in those mitochondrial-related diseases. Furthermore, as new mitochondrial therapies are developed (Fulda et al., 2010), noninvasive mapping of membrane potential may play a role in evaluation of response to therapy.

There is a long history of research on the measurement of ΔΨ_m_ dating back more than 50 years. Until about 10 years ago, work on ΔΨ_m_ was limited to bench-top experiments on cells (Rottenberg, 1979; Rottenberg, 1984), isolated mitochondria (Rottenberg, 1984; Wan et al., 1993), and isolated rat hearts (Kauppinen, 1983; Wan et al., 1993). This basic experimental approach often used a highly lipophilic cation, tetraphenylphosphonium labeled with tritium (^3^H-TPP^+^), which readily diffuses into cells and mitochondria (Kamo et al., 1979). When applied to isolated rat hearts, the ^3^H-TPP^+^ methodology is the forerunner to modern positron emission tomography (PET) scanning with lipophilic cations (Wan et al., 1993).

Due to the cellular and mitochondrial membrane potentials, the equilibrium concentration of TPP^+^ in cytosol is 3 to 10 times higher than in the extracellular space (ECS), and 100 to 500 times higher in the mitochondria compared to the ECS (Murphy, 2008). Measurements of the mitochondrial membrane potential have typically relied on compartment models of the TPP^+^ concentration, with the Nernst equation relating the concentrations on either side of a membrane to its electric potential (Nernst, 1889). A body of work has established ^3^H-TPP^+^ as the primary reference tracer method for measuring ΔΨ_m_. However, the use of ^3^H-tracers is not appropriate for human investigation because of its ionizing radiation, long physical half-life and attendant radiotoxicity. Moreover, for *in vivo* studies in man or animals, the lack of externally detectable radiation means that tissues must be biopsied and processed in order to use the ^3^H-TPP^+^ methodology.

To relax these limitations, we recently reported a method for quantitative *in vivo* PET mapping of the total (i.e., cellular plus mitochondrial) tissue membrane potential ΔΨ_T_, a proxy for ΔΨ_m_ (Alpert et al., 2018). We showed that our method, which relies on the measurement of the distribution volume of ^18^F-TPP^+^ using PET, provides measurements in normal and chronically injured pig myocardium that were consistent with prior work using ^3^H-TPP^+^. To extend the validation of the method, we now provide experimental evidence that (1) the distribution volume of ^18^F-TPP^+^ is sensitive to changes in ΔΨ_m_ and (2) that it is possible to observe mitochondrial depolarization during the period of the PET measurement. To facilitate this study, we used a specific type of intravenous input function (bolus plus constant infusion) designed to achieve a secular equilibrium of ^18^F-TPP^+^ during which we measure the concentration of ^18^F-TPP^+^ with PET. Then we perturbed the equilibrium with intracoronary infusion of a proton gradient uncoupler (Jastroch et al., 2014), BAM15 (Kenwood et al., 2014; To et al., 2019), that affects ΔΨ_m_ but not the cellular membrane potential.

The specific objectives of this study are to show: (1) coronary infusion of BAM15 causes the concentration of ^18^F-TPP^+^ to decrease over time in the corresponding vascular territory, and (2) to determine the BAM15 dose at which changes in PET concentration can be observed.

## Materials and Methods

### Theory

The volume of tracer distribution, V_T_, is measurable with PET (Perrier and Mayersohn, 1982). Assuming a compartmental tissue model for a PET voxel (Perrier and Mayersohn, 1982; Alpert et al., 2018), and the volume of distribution can be written as

$$V_{T}=\frac{{\bar{C}}_{PET}}{{\bar{C}}_{p}}=\left(1-f_{ecs}\right)$$

(1) $$\left(f_{mito}\cdot e^{-\beta \Delta \Psi_{T}}+\left(1-f_{mito}\right)\cdot e^{-\beta \Delta \Psi_{c}}\right)+f_{ecs}$$

where the experimental endpoints are $\bar{C_{\text{PET}}}$, the steady-state tissue concentration of ^18^F-TPP^+^ measured by PET, $\bar{C_{\text{p}}}$, the steady-state plasma concentration, *f*_ecs_, the tissue volume fraction occupied by the ECS, and *f*_mito_ the tissue volume fraction occupied by mitochondria. $\beta =\frac{zF}{RT}$ is a ratio of known physical constants – *F* is Faraday’s constant, *z* denotes the valence, *R* denotes the universal gas constant, and *T* symbolizes the temperature in degrees Kelvin. *f*_ecs_ is measured using ECG-triggered CT scans before and after administration of iodinated contrast media (see below). *f*_mito_ was set to 0.2568 mL/mL (Barth et al., 1992). See Alpert et al. (2018) for more details. There are several ways to measure V_T_. One way is to use a bolus injection of ^18^F-TPP^+^ and a dynamic PET acquisition protocol, followed by kinetic analysis of the measured activity concentration data (Alpert et al., 2018). Administering the tracer with intravenous bolus injection followed immediately by prolonged constant infusion allows an alternative approach requiring establishment of a secular equilibrium during which V_T_ can be measured as a simple tissue-to-plasma concentration ratio (Endres and Carson, 1998; Carson, 2000).

### Animal Welfare

Animals used in this study were housed and maintained under the supervision of the Massachusetts General Hospital (MGH), Institutional Animal Care and Use Committee (IACUC), and our study was conducted under a protocol approved by the MGH IACUC.

### Animal Preparation

Adult Yucatan minipigs were anesthetized with intramuscular telazol (4.4 mg/kg) and xylazine (2 mg/kg), tracheally intubated, and mechanically ventilated. Anesthesia was maintained with a mixture of isoflurane (1.5%) and oxygen. Once intubated and under general anesthesia, swine were positioned supine on the procedure table with a heating pad underneath. All procedures were performed using aseptic technique. Bilateral auricular dorsolateral veins were cannulated with a 22G angiocatheter for administration of IV fluids and medications. Heparin (150 units/kg) was administered intravenously prior to introducer sheath placement and additional heparin administered as needed to maintain activated clotting time (ACT) > 200 s. The femoral veins were cannulated percutaneously with 8F introducer sheaths for administration of TPP, CT, contrast, and venous blood sampling. The left femoral artery was cannulated percutaneously with a 6F introducer to monitor arterial blood pressure and blood gasses. A 7F introducer sheath was placed in the right femoral artery for introduction of a guide catheter. The 7F guide catheter was inserted through the introducer and advanced into the ostium of the main left coronary artery under fluoroscopic guidance. Angiographic images of the coronary artery were obtained from the standard 25 degree left anterior oblique view with a 10 degree cranial angle. Intracoronary BAM15 drug delivery was accomplished with a 0.92 mm over the wire Maverick Angioplastic PTCA catheter (Boston Scientific). The drug delivery catheter was advanced over a 0.36 mm guide wire through the 7F guide catheter. Under fluoroscopy, radio-opaque marker bands located at the tip of the catheter were used to position the catheter tip in the mid-left anterior descending (LAD) artery. Once drug delivery catheter position was confirmed by fluoroscopy, the 0.36 mm guide wire was withdrawn and the lumen used for intracoronary BAM15 delivery. Both catheters were anchored and fixed externally with sutures to avoid displacement during imaging.

Vascular access for cardiac hemodynamic monitoring was achieved via a 2 cm midline neck incision over the right side of the trachea. The right common carotid artery was identified and an 8F introducer sheath inserted via a guide wire into the lumen of the artery. Two surgical ties were positioned on either side of the introducer sheath for bleeding control. Under fluoroscopic observation, the 5F Transonic Scisense catheter was inserted through the 8FR introducer, advanced across the aortic valve via the aortic root, and placed into the left ventricular chamber.

### PET/CT Scanning

An intravenous bolus injection of ∼16 mCi of ^18^F-TPP^+^ followed immediately with an infusion of ^18^F-TPP^+^ (∼6 mCi in 60 mL, 0.33 mL/min over 180 min) were used for tracer administration. PET list mode data were acquired for 150 min beginning 30 min after the start of ^18^F-TPP^+^ bolus/infusion using a hybrid PET/CT system (GE Discovery MI; Pan et al., 2019). List mode data were framed as a dynamic series of 2 min image volumes. PET data were reconstructed using an OSEM algorithm with CT-based attenuation correction to yield radioactivity concentration maps in units of Bq/cc with 89 slices and a voxel size of 2.73 mm × 2.73 mm × 2.8 mm. Reconstructed PET image volumes were reoriented into the standard short axis projection, segmented as standard 17 segment polar maps according to the ACNC guidelines (Dilsizian et al., 2016), and used to form time activity curves (TACs). These TACs were analyzed during the secular equilibrium and inspected for evidence of depolarization during infusion of BAM15.

### Blood Sampling

Venous blood samples were drawn from the femoral vein in pigs 3 and 4 to estimate the ^18^F-TPP^+^ concentration during secular equilibrium. Beginning at 90 min after tracer administration and ending 30 min later, samples were drawn every 6 min.

### Measurement of Extracellular Space

Myocardial *f*_ECS_ was measured with a contrast-enhanced cardiac CT protocol consisting of three steps: First, a CT scan was performed to obtain baseline blood and myocardial attenuation in Hounsfield units (HU); second, a bolus of iodine contrast agent was administered intravenously (1.8 mgI/kg; 3 mL/sec) using a power injector followed by a saline flush; third, a repeat CT scan was performed to measure blood and myocardial HU after a standard 15-min delay, required for the contrast agent to reach equilibrium between myocardium and blood pools (Treibel et al., 2015; Lee et al., 2016). All cardiac CTs were acquired with prospective ECG gating (∼65–75% of R-R interval) and the following parameters: helical mode, tube voltage: 120 kVp; tube current: 200 mAs; gantry rotation time: 330 msec, matrix size: 512 × 512, voxel size: 0.70 mm × 0.70 mm × 5mm, and 100 slices. Non-local means filtering was applied to the reconstructed images to increase the image signal to noise ratio (Buades et al., 2005). The filtering parameters were: local patch size 3 × 3 × 3, search window 5 × 5 × 3. Next, the CT images were registered to the PET image (summation of late frames) to account for potential position mismatch between the CT and PET acquisitions. The pre- and post-contrast CT images were first rigidly aligned to the PET image, followed by elastic registration of the myocardium to refine alignment. The resulting deformation field was applied to both pre- and post-contrast CT images. All rigid and non-rigid registrations were performed using the open source software elastix (Klein et al., 2009).

Iodine contrast induces more absorption and scattering of x-ray, which results in increases in CT attenuation. In heart, intravascular contrast rapidly permeates the ECS. The ECS fraction was calculated as $f_{\text{ECS}}=\frac{\Delta HU_{\text{M}}}{\Delta HU_{\text{B}}}\times \left(1-Hct\right)$ where Δ*HU*_M_ and Δ*HU*_B_ represent the change in attenuation before and after contrast in the myocardium and in the blood, respectively, (Δ*HU* = Δ*HU*_post−contrast_−Δ*HU*_pre−contrast_), and Hct represents the hematocrit.

### Intracoronary Dose Response of BAM15

BAM15 was prepared with 3% DMSO in 10% P80. BAM15 dose response with respect to PET imaging was assessed in two minipigs: Beginning 50 min post injection/infusion of ^18^F-TPP^+^, BAM15 was infused into the LAD coronary artery and the dose was increased in three levels. PET data were reconstructed as 75 frames of 2 min each, reoriented into the standard short axis projection, segmented as standard 17 segment polar maps, and used to form TACs. These TACs were inspected for evidence of depolarization. Visualization of distinct local minima in the TACs following BAM15 infusion was considered evidence of depolarization. The first dose-response experiment began with very conservative BAM15 dose while the second study used higher BAM15 doses to demonstrate that an effect could be observed. Bam15 dosing is summarized in Table 1. Global values of myocardial ^18^F-TPP^+^ concentrations were used as the endpoint for assessing if changes could be attributed to BAM15. Blood sampling was not performed in the dose-response studies.

**TABLE 1:** Summary of BAM15 infusion dose and measurable effects on ^18^F-TPP^+^ concentration.

	Infusion BAM15 concentration (mg/min)	Total BAM15 infusion dose (mg)	Measurable effects on ^18^F-TPP^+^ concentration
Dosing study #1	0.01	0.15	None
	0.1	1.5	None
	0.5	7.5	Delayed, mild reversible decrease
Dosing study #2	1.0	25	Rapid, significant reversible decrease
	1.2	12	Rapid decrease and severe cardiac dysfunction
Study #3	1.0	25	Rapid, significant reversible decrease
Study #4	1.0	25	Rapid, significant reversible decrease

### Volume of Distribution and Membrane Potential

In pigs 3 and 4, the distribution of ^18^F-TPP^+^ was allowed to evolve for 100 min under constant infusion, leading to a secular equilibrium of ^18^F-TPP^+^ in the myocardium. No BAM15 was administered during this period. Data obtained in the epoch 95–100 min was used to measure $V_{\text{T}}=\frac{\bar{C_{\text{T}}}}{\bar{C_{\text{p}}}}$ and to estimate ΔΨ_T_. Infusion of BAM15 commenced at 100 min and continued until 125 min. In those cardiac segments showing a clear decrease in TPP^+^ concentration, we used *V*_T_ ≈ *f*_m_⋅(1−*f*_ECS_)*e*^−βΔΨ_T_^ as an approximation of Eq. 1 to estimate the value of V_T_ and ΔΨ_T_ in the period of lowest excursion, as well as for computation of parametric maps of ΔΨ_T_.

## Results

### Dosing Studies

**FIGURE 1:**
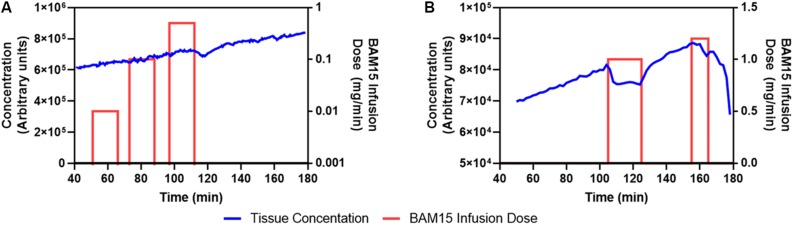
First **(A)** and second **(B)** BAM15 dosing study. Blue line denotes PET average concentration measurements of ^18^F-TPP^+^ in myocardium. The red bars indicate periods of BAM15 intracoronary infusion. The height of the bar is proportional to the BAM15 dose rate and is keyed to the dose rate scale on the right side of the graph. Cumulative BAM15 dose is obtained by multiplying the dose rate by the infusion time. A putative dose response is noted beginning at about 115 min in A; whereas, B suggests that the BAM15 infusion causes depolarization of mitochondria during the period 110–125 min. After the first BAM15 infusion was stopped, the ^18^F-TPP^+^ concentration resumed is original trajectory.

As shown in Figure 1A, during constant intravenous infusion of ^18^F-TPP^+^, the myocardial ^18^F-TPP^+^ concentration rose nearly linearly between 40 and 95 min while the BAM15 infusion rate was increased ten-fold, from 0.01 mg/min (total 0.15 mg) to 0.1 mg/min (total 1.5 mg), with no measurable effects of BAM15. At 95 min the BAM15 dose was increased to 0.5 mg/min (total 7.5 mg) and the PET concentration decreased about 20 min after the beginning of BAM15 infusion.

Figure 1B shows the average myocardial TAC of the second dosing study. The BAM15 infusion rate was set at 1 mg/min and infused into the LAD artery over a 25 min period beginning at 100 min after the PET infusion began. The global ^18^F-TPP^+^ myocardial concentration immediately decreased and a new secular equilibrium was observed in the period 110–120 min. At the end of the first BAM15 infusion the concentration of ^18^F-TPP^+^ resumed a roughly linear increase until 155 min when the second BAM15 infusion started. The second infusion of 1.2 mg/min was terminated after 10 min due to cardiac dysfunction. The cumulative BAM15 dose before end of study was 37 mg.

### Effect of BAM15

**FIGURE 2:**
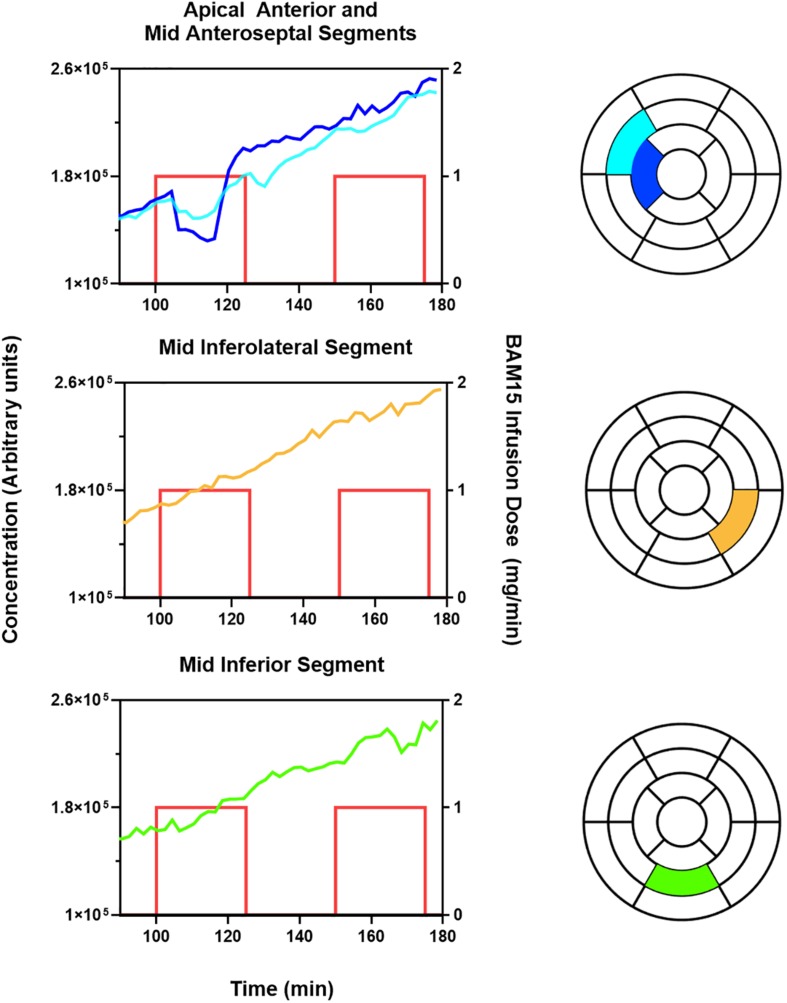
BAM15 infusion in left anterior descending (LAD) artery causes depolarization of myocardium in corresponding vascular territory, with less effect in left circumflex (LCx), and right coronary artery (RCA). The blue and aqua colored lines index the ^18^F-TPP^+^ concentration versus time for specific cardiac segments corresponding to the LAD territory as indicated by the text above each graph. The yellow and green colored lines index the ^18^F-TPP^+^ concentration versus time for specific cardiac segments corresponding to the LCx and RCA territory, respectively. The standard 17 segment polar map of the myocardium is also provided for each graph, to help the reader visualize the location of the plotted segments. The red lines denote the times during which BAM15 was infused; the height of the lines indicate the infusion dose rate.

Figure 2 demonstrates that BAM15 infusion into the mid LAD artery causes a decrease in the concentration of ^18^F-TPP^+^ in the corresponding vascular territory. The top panel presents TACs for apical anterior and mid anteroseptal myocardium, segments 8 and 14 of the standard 17 segment polar model. Shortly after start of BAM15 infusion into the LAD, concentration of ^18^F-TPP^+^ decreased, and remained depressed for about 10 min before beginning an upward trajectory. A second BAM15 infusion, begun 25 min later, demonstrated a much weaker response in these segments. Considering the whole myocardium, the response to BAM15 varied from segment to segment; greatest in apical segment 14 and least in segments 10 and 11 which are not parts of the LAD territory. These results are consistent with the placement of the catheter midway in the LAD artery and support the idea that it is possible to monitor, in real time, changes in ^18^F-TPP^+^ concentration, as an indicator of mitochondrial membrane depolarization.

**FIGURE 3:**
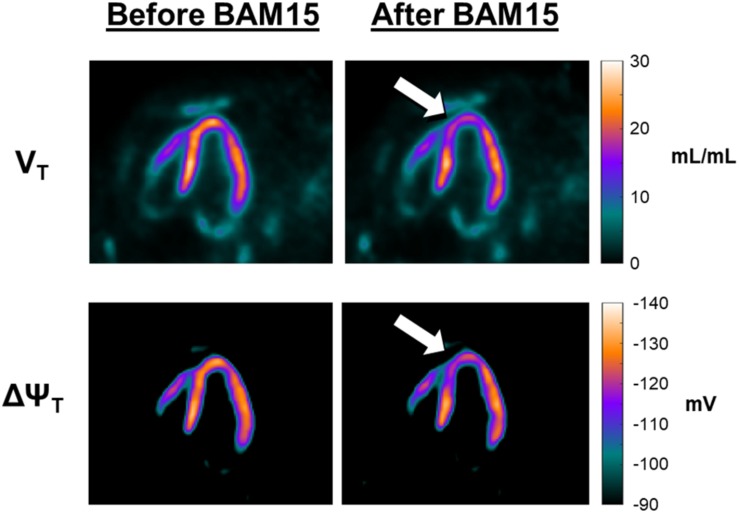
Effect of BAM15 on ^18^F-TPP^+^ volume of distribution (V_T_; top) and tissue membrane potential (ΔΨ_T_; bottom). Shown are vertical long axis projections of the pig heart, with the apex facing the top of page. The images in the left column are from data acquired before infusion of BAM15. Images on the right were obtained during a secular equilibrium during BAM15 infusion. The color bars, to the right of each row, provide a quantitative index of the parameters. The white arrows serve to guide the eye toward areas of the myocardium depolarized by BAM15 infusion. Intracoronary infusion of BAM15 in the left anterior descending artery reduces the V_T_ of the tracer in apical and apical septal segments of the myocardium. Corresponding depolarization of the ΔΨ_T_ is demonstrated by comparison of the before and after BAM15 images.

Figure 3 provides a more detailed quantitative analysis, comparing data obtained at secular equilibrium with and without BAM15. Parametric images of V_T_ and ΔΨ_T_, are shown in the vertical long axis projection, allowing examination of effects in apex and septum. Color/intensity bars on the right side of Figure 3 provide a quantitative index of the parameter values. Before the infusion of BAM15 the parametric images of V_T_ and ΔΨ_T_ are similar to those obtained in our earlier study (Alpert et al., 2018), with values of V_T_ about 23 mL/mL and ΔΨ_T_ near -133 mV. Comparison with the “after BAM15” images shows that V_T_ decreased to about 18 mL/mL and ΔΨ_T_ fell to about -119 mV. In other word, assuming that BAM15 did not affect the cellular membrane, there was about 10 mV depolarization of mitochondrial membrane potential attributable to BAM15 uncoupling of the proton current.

## Discussion

The rationale for this study is to provide direct evidence that ^18^F-TPP^+^ can be used to assess acute depolarization of myocardial mitochondria. This work is an extension of our initial report (Alpert et al., 2018) which provided the first *in vivo* maps of total myocardial membrane potential in normal and chronically injured pig myocardia. In the case of chronic macroscopic tissue injury, there is probably a mixture of mitochondrial states, those with complete collapse of mitochondrial membrane potential and cell death, others with partial depolarization, and still others that are essentially normal. However, for applications such as early detection of cardiotoxicity, the ability to detect partial depolarization of myocardial mitochondria may be important for managing such injuries before depolarization leads to irreversible injury (McCluskey et al., 2019).

As noted above, BAM15 is a novel protonofore uncoupler, thought to be equipotent to carbonyl cyanide p-trifluoromethoxyphenylhydrazone (FCCP), that partially depolarizes the inner mitochondrial membrane but not the plasma membrane. The article by Kenwood et al. (Kenwood et al., 2014), mentions I.P. injection of BAM15 in mice but gives no dosing guidance for constant intracoronary infusion in large animals, nor does the literature discuss the total body distribution of BAM15, its kinetics or its metabolism. This study provides the first attempt to fill that gap, but it must be said that in this regard our work was expedient; our goal was simply to adjust the dose of BAM15 to produce an observable effect on the concentration history of ^18^F-TPP^+^ in pig myocardium. In addition to the direct effects noted in heart muscle, it is likely that circulation of BAM15 causes similar effects in other tissues, such as liver and skeletal muscle. Investigating these effects was beyond the scope of this report.

Before starting this work, we did not have any prediction for the magnitude of depolarization that might result from infusion of BAM15. But Sack (2006) has reviewed the mitochondrial depolarization and the role of uncoupling proteins in ischemia tolerance, suggesting the maximal reduction of mitochondrial membrane potential with robust overexpression of the protonofore uncoupler UCP2 approaches 15 mV. Thus, it is interesting that in this study, despite direct coronary infusion of 1 mg/min over 25 min, that we found modest depolarization of mitochondria in proximity of the catheter in the mid-LAD artery with maximum depolarization of about -10 mV.

The basic results of this study, observation of reduction in the concentration of a highly lipophilic cation, ^18^F-TPP^+^ in response to BAM15 infusion, are well supported by the data. However, there was considerable variation in the strength and location of response that cannot be fully evaluated with the small sample size of this study. Nevertheless, we can speculate that the variation in the effective catheter position in the LAD can explain much of the variation. On the other hand, there were cases in which the first BAM15 infusion provoked a strong depolarization whereas the second infusion did not produce an observable effect at the same location. We have no explanation for those findings. Finally, no control infusions were performed and DMSO and P80 could be involved in the observed changes of ^18^F-TPP^+^ concentration. However, in the first dosing study, no change in ^18^F-TPP^+^ concentrations was observed with the first two infusion of BAM15, which had same infusion rate to those producing changes (1 mL/min), suggesting that the effects observed at higher doses of BAM15 were not related to the solvents DMSO and P80.

## Conclusion

This pilot study demonstrates that it is possible to observe acute mitochondrial depolarization *in vivo* using PET imaging, thus confirming that our TPP^+^-PET methodology is sensitive to mitochondrial membrane potential. Intracoronary infusion of 1 mg/min of BAM15 over 25 min leads to measurable and transient depolarization of ∼10 mV. *In vivo* imaging of myocardial membrane potential with ^18^F-TPP^+^ holds great potential in research and clinical applications.

## Data Availability Statement

The datasets generated for this study are available on request to the corresponding author.

## Ethics Statement

The animal study was reviewed and approved by the Institutional Animal Care and Use Committee of the Massachusetts General Hospital. Animals used in this study were housed and maintained under the supervision of the Massachusetts General Hospital (MGH) Institutional Animal Care and Use Committee (IACUC) and our study was conducted under a protocol approved by the MGH IACUC.

## Author Contributions

NA contributed to the conceptualization of the study, data analysis, and writing of the original draft. MP-G contributed to the conceptualization of the study, data acquisition and analysis, and writing of the original draft. SK, KR-T, and YP contributed to the data acquisition and analysis as well as the review and editing of the manuscript. TS contributed to the data analysis. MN and GE contributed to the conceptualization of the study and the review and editing of the manuscript.

## Conflict of Interest

The authors declare that the research was conducted in the absence of any commercial or financial relationships that could be construed as a potential conflict of interest.
